# Sub-chronic toxicopathological study of lantadenes of *Lantana camara* weed in Guinea pigs

**DOI:** 10.1186/s12917-018-1444-x

**Published:** 2018-04-13

**Authors:** Rakesh Kumar, Rinku Sharma, Rajendra D. Patil, Gorakh Mal, Adarsh Kumar, Vikram Patial, Pawan Kumar, Bikram Singh

**Affiliations:** 1 Disease Investigation Laboratory, ICAR-Indian Veterinary Research Institute, Regional Station, Palampur, Himachal Pradesh India; 2 DGCN COVAS, CSK HPKV, Palampur, Himachal Pradesh India; 3 CSIR-IHBT, Palampur, Himachal Pradesh India

**Keywords:** Lantadenes, Sub-chronic toxicity, Haematology, Serum markers, Pathology, Oxidation stress, Guinea pigs

## Abstract

**Background:**

In the field conditions, animals regularly consume small quantities of lantana leaves either while grazing or due to mixing with regular fodder.

The hypothesis of this study was that consumption of lantana toxins over a long period of time leads to progression of sub-clinical disease.

Toxicopathological effects of sub-chronic (90 days) administration of lantadenes of *L. camara* were investigated in guinea pigs. For this, a total of 40 animals were divided into 5 groups whereby groups I, II, III and IV were orally administered lantadenes, daily at the dose of 24, 18, 12, and 6 mg/kg bw, respectively while group V was control. The animals were evaluated by weekly body weight changes, haematology, serum liver and kidney markers, tissue oxidative markers and histopathology.

**Results:**

The results of significant decrease in weekly body weights, haematology, liver and kidney marker enzymes (alanine aminotransaminase, aspartate aminotransaminase, acid phosphatase and creatinine), oxidation stress markers (lipid peroxidation, reduced glutathione, superoxide dismutase and catalase) in liver and kidneys, histopathology, and confirmation of fibrous collagenous tissue proliferation by Masson’s Trichome stain showed that lantadenes led to a dose-dependent toxicity in decreasing order with the highest dose (24 mg/kg bw) producing maximum lesions and the lowest dose (6 mg/kg bw) producing minimum alterations.

**Conclusions:**

The study revealed that lantadenes which are considered to be classical hepatotoxicants in acute toxicity produced pronounced nephrotoxicity during sub-chronic exposure. Further studies are needed to quantify the levels of lantadenes in blood or serum of animals exposed to lantana in field conditions which would help to assess the extent of damage to the vital organs.

## Background

*Lantana camara* is a noxious weed growing in tropical and subtropical parts of the world [1]. *L. camara* toxicity caused by lantadenes is characterized by intrahepatic cholestasis, liver damage and photosensitization. Both ruminants including cattle, sheep, buffalo, goats and non-ruminants like horses, guinea pigs, rabbits, female rats are susceptible to lantana toxicity. Guinea pigs exhibit most typical signs comparable to experimental or field cases of ruminants affected with lantana toxicity. So, guinea pigs are often used as a preferred animal model for lantadene toxicity studies. The leaves of red flower variety (*L. camara* var. *aculeata*) of lantana are mainly toxic to animals and contain pentacyclic triterpenoids [2, 3]. Lantadene A (LA) and lantadene B (LB), lantadene C (LC) and lantadene D (LD) are present in major quantity while reduced lantadene A (RLA) and reduced lantadene B (RLB) are the minor components having lesser importance as compared to other constituents present [4, 5]. Among these compounds, LA is the most hepatotoxic component while others are of little importance [2].

Generally this plant is not used as a fodder, but in field conditions, the outbreaks of lantana poisoning are seen during fodder scarcity, drought and flood where animals may consume small quantities of lantana leaves either while grazing or due to mixing with regular fodder. The consumption of lantana toxins over a long period of time may lead to progression of sub-clinical disease followed by death of the animal. However, the cause of death in such cases remains obscured due to complete lack of information about the toxicopathological effects which could be induced by sub-chronic ingestion of lantana leaves in animals. Any specific treatment for lantana toxicity is not available however some conventional therapies like fluid therapy, activated charcoal (5 g/kg), tefroli powder, liv-15 etc. can be used. The present study was conducted to investigate whether sub-chronic ingestion of lantadenes would lead to any adverse effects in guinea pig laboratory animal model.

## Methods

### Procurement, maintenance and housing of experimental animals

Forty guinea pigs weighing approximately 200–300 g and of either sex were procured from Laboratory Animal Resource Section, ICAR-IVRI, Izatnagar for the experimental study. All the animals were examined for any ailment or abnormality and their body weights were recorded. The animals were maintained in the Laboratory Animal Housing Facility of ICAR-IVRI Regional Station, Palampur. All the guinea pigs were kept in polypropylene cages and provided with 12 h light/dark cycle, temperature (23 ± 2 °C) and humidity (55 ± 10% RH). The experimental protocols were reviewed and approved by the Institutional Animal Ethics Committee (No. PLP-IAEC 8). All sanitary and hygienic measures were observed as per the CPCSEA guidelines. The animals were provided ad libitum access to standard laboratory animal diet supplemented with vitamin C (Limcee, 1000 mg/kg feed) and clean water during the experimental trial.

### Collection, processing and isolation of lantadenes from *L. camara* leaves

Leaves of red flower variety of *L. camara* var. *aculeate* were collected during the month of August–September from an area adjoining Palampur town located at an altitude of 1200 m above mean sea level. The samples were oven dried at 55 °C and ground to a fine powder of 1 mm particle size. The extraction of lantadenes was carried out by protocol described by Parimoo and co-workers [6]. The isolated lantadenes were stored in sealed vials at room temperature until further use. The purification was monitored by thin layer chromatography (TLC). The quantification and characterization of lantadenes was done with the help of reversed-phase HPLC. The TLC and reversed-phase HPLC analysis protocols were as described earlier [6].

### Experimental study

The animals were provided with an acclimatization period of 7 days. All the animals were weighed and divided into 5 groups with 8 animals (4 males and 4 females) in each group. The experimental animals (group I, II, III and IV) were administered graded doses of lantadenes, orally, in gelatin capsules, once daily for 90 days. The dose selection was made on the basis of previous study on sub-acute toxicity of *L. camara* by Parimoo and co-workers [7]. An interval of one hour was kept between administration of lantadenes and feed. Group V was control group and it received normal feed and water. All the animals were euthanized using chloroform at day 90 and different samples for laboratory analysis were collected. During euthanasia, sufficient ventilation and a means of exhausting waste gases in the closed container was made.

### Collection of blood and separation of serum

Approximately 4 ml of blood was drawn from posterior vena cava in glass tubes and kept at room temperature for 2–4 h in slanting position, followed by centrifugation at 2000 rpm for 15 min to collect maximum quantity of serum. The collected serum was then stored in screw cap vials and kept at –70 °C for further estimation of various serum biochemical parameters.

### Serum biochemical analysis

Serum biochemicals such as alanine aminotransferase (ALT), aspartate aminotransferase (AST), alkaline phosphatase (ALP), bilirubin, creatinine and total protein were estimated using commercial kits (Span Diagnostic Ltd., India) by employing automatic biochemistry analyzer (Bayer RA 50). The manufacturer’s protocol was followed for all the estimations. However, acid phosphatase (ACP) was determined by the method described by Bergmeyer [8] and was expressed in IU/L.

### Oxidation stress determination

Viable tissues samples of liver and kidneys were collected aseptically in sterile, screw capped polypropylene vials using sterile scissors and forceps, transported on ice and stored at -70 °C after proper labeling till further processing.

### Preparation of homogenate

Tissue samples of liver and kidneys were homogenized (Remi, 12 U-56) in the ratio of 1:10 (200 mg tissue in 2 ml PBS) in ice cold 0.1 M PBS (pH 7.4). The homogenate was centrifuged for 10 min at 10,000 rpm. The supernatant was used for the estimation of superoxide dismutase, catalase, lipid peroxidation, reduced glutathione and total protein.

### Lipid peroxidation

Lipid peroxidation (LPO) was estimated by the method described by Dawra and co-workers [9]. The absorbance was read at 535 nm. Results were calculated from ∆E using molar extinction coefficient of 1.56 × 10^5^/M/cm. The results were expressed as μmoles of MDA (malondialdehyde) production per g of wet tissue.

### Reduced glutathione

Reduced glutathione (GSH) was estimated by the method described by Sedlak and Lindsay [10]. The absorbance was measured at 412 nm. Calculation was done by using extinction co-efficient (EC = 13,100/M/cm) and results were expressed in nM/g of wet tissue.

### Catalase

Catalase enzyme was estimated by the method of Aebi [11]. The decrease in absorbance was monitored at 240 nm. The results were expressed as k/mg protein where k stands for nano moles of H_2_O_2_ utilized/min.

### Superoxide dismutase

Superoxide dismutase (SOD) enzyme was estimated by the method described by Nishikimi and co-workers [12]. The increase in absorbance was measured at 560 nm. The unit of superoxide dismutase was defined as the amount of enzyme causing 50% inhibition of the rate of reduction of nitro blue tetrazolium. The results were expressed as units/mg protein.

### Protein estimation

The protein content in the homogenate was estimated by Lowry and co-workers [13]. Bovine serum albumin (BSA) (1 mg/ml, Lobachemie) was used as standard. The absorbance of blue colour developed was recorded at 578 nm**.** The protein content in the sample was calculated from standard curve prepared using different concentrations of BSA.

### Gross and histopathological examination

A detailed necropsy examination was conducted on animals and the gross findings were recorded. Neutral buffered formalin fixed tissues (liver, kidneys, heart, brain, spleen, lymph nodes, stomach, intestine etc.) were subjected to histopathological processing and staining as per standard procedures [14]. Haematoxylin and Eosin (HE) stained individual sections were microscopically examined and the histopathological alterations were recorded and digitally photomicrographed (Olympus BX53). The histopathological lesions in liver and kidneys were graded with ordinal score of 0, no change; 1, mild (< 25% organ affected); 2, moderate (26–50% organ affected); and 3, severe (51–75% organ affected); which reflects the changes of low, medium and high grades, respectively [15]. The degree of fibrosis in liver and kidneys were assessed as per Scheuer/Batts, Ludwig/Tsui fibrosis scoring system, where the stages 0, 1, 2, 3, 4 indicates no fibrosis, portal/peri-portal fibrosis, septal fibrosis and cirrhosis, respectively [16].

### Masson’s Trichome stain (MST)

Parallel sections of tissues showing fibrous tissue proliferations in HE were stained with special stain, Masson’s trichome stain [14].

### Statistical analysis

One-way analysis of variance (ANOVA) was used to detect differences among groups and the means were compared by Dunnett’s Multiple Comparison test using critical difference at 5% level of significance (*P* ≤ 0.05). All analyses were performed with Graph Pad InStat software (San Diego, USA). Kruskal-Wallis Test (Non-parametric ANOVA) was used for analysis of microscopic scoring in liver and kidneys.

## Results

**Fig. 1:**
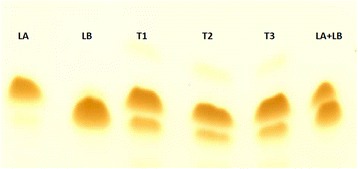
Thin layer chromatogram of lantadenes isolated from *Lantana camara* Linn. var. *aculeate* leaves. LA: lantadene A standard; LB: lantadene B standard; T1, T2 & T3: Triplicates of test sample; LA + LB: Mixture of lantadene A standard+ lantadene B standard

**Fig. 2:**
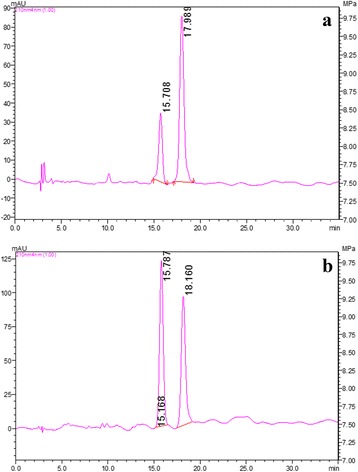
**a** HPLC chromatogram of test lantadene A and B at 210 nm. **b** HPLC chromatogram of lantadene A and B standard mixture at 210 nm. [Total lantadene content = 62.27%; (49.18% LA + 13.09% LB)]

Approximately 650 g of green lantana leaves yielded 100 g of dried leaf powder from which 490.20 mg lantadenes were obtained by the protocol described earlier [6]. In the present study, the LA and LB content in the leaves was estimated to be 49.18% and 13.09%, respectively by reversed phase-HPLC (Figs. 1 and 2a and b).

### Weekly body weight gain

A significant decline in the body weights of animals of group I was observed as compared with the animals of control group (Table 1).

**Table 1:** Weekly body weight (g) (Mean ±SE) of different treatment groups during sub-chronic *L. camara* toxicity study in guinea pigs

	GI	GII	GIII	GIV	GV
Day −0	241.17 ± 7.93^a^	250.17 ± 12.52^a^	260.33 ± 8.09^a^	256.17 ± 12.24^a^	268.50 ± 10.97^a^
Day −7	253.50 ± 11.87^a^	258.67 ± 14.21^a^	271.17 ± 8.56^a^	272.50 ± 12.59^a^	288.00 ± 15.91^a^
Day −14	262.83 ± 13.53^a^	277.17 ± 15.78^a^	277.33 ± 14.93^a^	275.67 ± 19.79^a^	307.17 ± 17.92^a^
Day −21	268.17 ± 10.34^a^	279.83 ± 8.02^a^	281.33 ± 22.86^a^	283.67 ± 26.31^a^	320.00 ± 24^a^
Day −28	256.33 ± 26.56^a^	284.50 ± 21.37^a^	296.83 ± 41.27^a^	296.00 ± 30.20^a^	337.83 ± 24.38^a^
Day −35	258.33 ± 18.38^a^	279.33 ± 24.12^a^	291.00 ± 32.75^a^	294.17 ± 31.73^a^	346.67 ± 22.23^a^
Day −42	263.50 ± 25.69^a^	278.17 ± 36.08^a^	287.00 ± 40.51^a^	292.50 ± 37.17^a^	368.17 ± 22.30^a^
Day −49	267.17 ± 17.27^a^	285.50 ± 42.69^a^	288.00 ± 42.40^a^	304.17 ± 36.12^a^	376.17 ± 22.75^a^
Day −56	276.00 ± 23.94^a^	283.00 ± 40.17^a^	290.17 ± 39.73^a^	341.00 ± 45.68^a^	385.17 ± 25.11^a^
Day −63	277.00 ± 13.46^a^	297.83 ± 32.11^ab^	327.83 ± 32.52^ab^	337.17 ± 37.41^ab^	395.00 ± 16.25^b^
Day −70	288.83 ± 13.97^a^	299.67 ± 32.36^ab^	342.33 ± 39.98^ab^	369.33 ± 41.95^ab^	403.83 ± 16.43^b^
Day −77	298.00 ± 26.31^a^	313.67 ± 39.98^ab^	354.33 ± 29.03^ab^	374.33 ± 25.09^ab^	409.50 ± 16.70^b^
Day −84	313.50 ± 20.86^a^	327.17 ± 19.73^ab^	356.17 ± 31.91^ab^	383.33 ± 27.46^ab^	415.17 ± 24.37^b^
Day −90	312.50 ± 15.94^a^	333.33 ± 35.11^ab^	347.17 ± 34.80^ab^	389.33 ± 25.04^ab^	425.33 ± 26.72^b^

^a-b^Values within rows with different superscripts differ significantly by ANOVA (*P* ≤ 0.05). GI = Lantadenes @ 24 mg/kg bw; GII = Lantadenes @ 18 mg/kg bw; GIII = Lantadenes @ 12 mg/kg bw; GIV = Lantadenes @ 6 mg/kg bw; GV = control group, *n* = 6

### Haematology

There was a significant decrease in Hb and PCV levels of animals of group I (12.18 ± 0.55 g/dl and 36.28 ± 2.07%, respectively) as compared to group V (control). Total platelet counts of groups I (451.83 ± 55.03 × 10^3^/μl) and II (464.5 ± 60.24 × 10^3^/μl) were significantly decreased as compared to control (677 ± 58.26 × 10^3^/μl). The results of haematological changes have been presented in Table 2.

**Table 2:** Hematological values (Mean ± SE) of different groups during sub-chronic *L. camara* toxicity in guinea pigs

	GI	GII	GIII	GIV	GV
TLC (×10^3^/μl)	8.82 ± 0.61	8.52 μ ± 1.18	7.67 ± 1.85	7.55 ± 2.03	6.73 ± 0.51
TEC (× 10^6^/μl)	5.583 ± 0.34	5.59 ± 0.18	5.87 ± 0.18	5.93 ± 0.21	6.03 ± 0.12
Hb (g/dl)	12.18 ± 0.55^a^	12.52 ± 0.65^ab^	12.68 ± 0.54^ab^	13.16 ± 0.47^ab^	14.31 ± 0.33^b^
PCV (%)	36.28 ± 2.07^a^	39.3 ± 2.51^ab^	43.07 ± 2.28^ab^	44.87 ± 1.90^ab^	45.59 ± 1.06^b^
MCV (fl)	69.22 ± 2.89	71.26 ± 2.08	72.22 ± 0.62	74.83 ± 1.55	75.63 ± 0.74
MCH (pg)	20.92 ± 1.30	21.913 ± 0.85	23.23 ± 0.58	23.22 ± 0.51	23.74 ± 0.13
MCHC (g/dl)	29.62 ± 1.02	29.72 ± 1.18	31.13 ± 0.78	31.33 ± 0.30	31.59 ± 0.26
Platelet Count (×10^3^/μl)	451.83 ± 55.3^a^	464.5 ± 60.24^a^	596.5 ± 50.30^ab^	623.17 ± 44.29^ab^	677 ± 58.26^b^
Heterophil Count (×10^3^/μl)	2.19 ± 0.15	3.65 ± 0.56	3.43 ± 0.36	3.39 ± 0.56	2.28 ± 0.36
Lymphocyte Count (×10^3^/μl)	5.48 ± 0.44	4.32 ± 0.50	4.09 ± 0.69	3.98 ± 0.63	4.03 ± 0.34
Monocyte Count (×10^3^/μl)	0.48 ± 0.11	0.42 ± 0.05	0.37 ± 0.07	0.3 ± 0.12	0.34 ± 0.04
Eosinophil Count (×10^3^/μl)	0.32 ± 0.16	0.45 ± 0.23	0.32 ± 0.17	0.38 ± 0.20	0.48 ± 0.22
Basophil Count (×10^3^/μl)	0.03 ± 0.01	0.02 ± 0.00	0.02 ± 0.01	0.01 ± 0.00	0.02 ± 0.00
Heterophil (%)	27.75 ± 2.18	37.2 ± 5.40	36.13 ± 4.27	37.08 ± 4.67	31.39 ± 5.06
Lymphocyte (%)	63.27 ± 2.89	53.98 ± 6.25	55.63 ± 3.45	54.51 ± 4.49	58.75 ± 5.36
Monocyte (%)	5.75 ± 0.94	5.55 ± 0.76	5.07 ± 0.53	4.95 ± 0.84	5.25 ± 0.40
Eosinophil (%)	2.93 ± 1.52	3 ± 0.99	2.93 ± 1.49	3.33 ± 1.12	4.39 ± 1.16
Basophil (%)	0.3 ± 0.07	0.27 ± 0.03	0.23 ± 0.06	0.13 ± 0.03	0.23 ± 0.06

^a-b^Values within rows with different superscripts differ significantly by ANOVA (*P* ≤ 0.05). GI = Lantadenes @ 24 mg/kg bw; GII = Lantadenes @ 18 mg/kg bw; GIII = Lantadenes @ 12 mg/kg bw; GIV = Lantadenes @ 6 mg/kg bw; GV = Control group, *n* = 6

### Serum biochemical estimation

AST (107.07 ± 8.64 IU/L) levels of animals of group I were significantly (*P* < 0.05) elevated as compared to control group. However, there was no significant difference in AST values amongst groups II (100.03 ± 10.77 IU/L), III (91.08 ± 19.03 IU/L), IV (80.15 ± 15.14 IU/L) and V (58.10 ± 7.58 IU/L). ALT (77.82 ± 8.65 IU/L) levels of animals of group I were significantly elevated as compared to control group. But there was no significant difference in ALT values amongst groups II (63.88 ± 4.28 IU/L), III (62.32 ± 6.79 IU/L), IV (55.72 ± 7.30 IU/L) and V (52.14 ± 6.60 IU/L). There was no significant difference in ALP levels amongst different groups as compared to control. ACP (111.20 ± 15.30 IU/L) levels of animals of group I showed significant elevation as compared to control group. However, there was no significant difference in ACP values amongst groups II (90.74 ± 14.74 IU/L), III (75.96 ± 20.77 IU/L), IV (67.05 ± 14.13 IU/L) and V (43.83 ± 14.4 IU/L). The values of bilirubin were unchanged in all treatment groups as compared to control. Creatinine levels of animals of groups I (1.21 ± 0.22 mg/dl) and II (1.14 ± 0.17 mg/dl) were significantly increased as compared to control. However, creatinine values of groups III (0.99 ± 0.10 mg/dl), IV (0.75 ± 0.10 mg/dl), and V (0.54 ± 0.07 mg/dl), did not exhibit any significant difference. Serum protein levels of group IV and V did not exhibit any significant difference. While serum protein levels of animals of groups I (4.05 ± 0.27 g/dl), II (4.42 ± 0.45 g/dl) and III (4.52 ± 0.49 g/dl) were significantly decreased as compared to control (6.60 ± 0.42 g/dl). The results of various biochemical parameters have been shown in Table 3.

**Table 3:** Biochemical values (Mean ± SE) in different groups during sub-chronic *L. camara* toxicity in guinea pigs

GROUP	AST (IU/L)	ALT (IU/L)	ALP (IU/L)	ACP (IU/L)	Protein (g/dl)	Bilirubin (mg/dl)	Creatinine (mg/dl)
GI	107.07 ± 8.64^a^	77.82 ± 8.65^a^	116.60 ± 16.96^a^	111.20 ± 15.30^a^	4.05 ± 0.27^a^	1.01 ± 0.15^a^	1.21 ± 0.22^a^
GII	100.03 ± 10.77^ab^	63.88 ± 4.28^ab^	105.90 ± 17.71^a^	90.74 ± 14.74^ab^	4.42 ± 0.45^a^	0.84 ± 0.07^a^	1.14 ± 0.17^a^
GIII	91.08 ± 19.03^ab^	62.32 ± 6.79^ab^	100.48 ± 15.8^a^	75.96 ± 20.71^ab^	4.52 ± 0.49^a^	0.76 ± 0.16^a^	0.99 ± 0.10^ab^
GIV	80.15 ± 15.14^ab^	55.72 ± 7.30^ab^	98.69 ± 13.48^a^	67.05 ± 14.13^ab^	5.21 ± 0.41^ab^	0.73 ± 0.21^a^	0.75 ± 0.10^ab^
GV	58.10 ± 7.58^b^	52.14 ± 6.60^b^	78.03 ± 14.8^a^	43.83 ± 14.4^b^	6.60 ± 0.42^b^	0.49 ± 0.11^a^	0.54 ± 0.07^b^

^a-b^Values within columns with different superscripts differ significantly by ANOVA (*P* ≤ 0.05). GI = Lantadenes @ 24 mg/kg bw; GII = Lantadenes @ 18 mg/kg bw; GIII = Lantadenes @ 12 mg/kg bw; GIV = Lantadenes @ 6 mg/kg bw; GV = control group, *n* = 6

### Oxidation stress estimation

Malondialdehyde is considered as a marker of free radical-mediated lipid peroxidation injury. So, increased level of MDA lead to the failure of free-radical scavenging mechanisms. In the present study, LPO levels in liver of animals of almost all groups (II, 1.16 ± 0.16; III, 0.88 ± 0.260; IV, 0.74 ± 0.17; V, 0.73 ± 0.11) were increased but the increase was significant (*P* < 0.05) only in animals of group I (1.50 ± 0.23 μM of MDA/g wet tissue). MDA levels in kidneys of lantadene administrated animals were significantly increased in animals of group I (1.78 ± 0.34 μM of MDA/g wet tissue) as compared to the control (0.64 ± 0.09 μM of MDA/g wet tissue), while this elevation was not significant in other groups. Superoxide dismutase (SOD) has been reported as one of the most important enzyme in enzymatic antioxidant defence system. It scavenges superoxide anion to form H_2_O_2_, hence diminishes the toxic effects induced by this free radical. In the present study, there was a significant decrease in SOD values in the liver of animals of groups I (4.82 ± 0.38 U/mg protein) (24 mg/kg bw) and II (6.04 ± 0.50 U/mg protein) (18 mg/kg bw) as compared to the control group (7.58 ± 0.27 U/mg protein). Similar pattern of decrease in SOD levels was seen in the kidneys of the animals of groups I (3.94 ± 0.16 U/mg protein) and II (4.46 ± 0.31 U/mg protein) as compared to the control group (6.76 ± 0.66 U/mg protein). Reduced glutathione (GSH) is one of the most abundant non-biological antioxidant present in liver. GSH levels in liver homogenate of animals of groups I (19.12 ± 1.29 nM/g of wet tissue) and II (24.04 ± 1.90 nM/g of wet tissue) were significantly decreased as compared to control group. Reduction of this enzyme in kidney homogenate was only seen in the animals of group I.

Catalase decomposes H_2_O_2_ and protects the tissues from highly reactive hydroxyl radicals. Reduction in the anti-oxidative activities of this enzyme may result in a number of deleterious effects due to accumulation of superoxide radicals and H_2_O_2_. In the present study, the catalase levels were significantly lower in the liver (14.27 ± 1.62 K/mg protein) as well as kidneys (12.83 ± 0.46 K/mg protein) of animals of group I. The protein levels in liver homogenate were significantly decreased in animals of group I as compared to control, while the levels in other groups did not differ significantly from control. The protein levels in kidney homogenate were also decreased in animals of groups I and II as compared to control. The values of oxidation stress in liver and kidney homogenate have been presented in Tables 4 and 5, respectively.

**Table 4:** Lipid peroxidation, catalase, superoxide dismutase, reduced glutathione, and protein values (Mean ± SE) in tissue homogenate of liver during sub-chronic *L. camara* toxicity in guinea pigs

Group	LPO (μM of MDA/g wet tissue)	Catalase (K/mg protein)	Superoxide dismutase (U /mg protein).	Reduced glutathione (nM/ g of wet tissue)	Protein (mg/dl)
GI	1.50 ± 0.23^a^	14.27 ± 1.62^a^	4.82 ± 0.38^a^	19.12 ± 1.29^a^	50.81 ± 5.70^a^
GII	1.16 ± 0.16^ab^	20.01 ± 3.15^ab^	6.04 ± 0.50^ab^	24.04 ± 1.90^ab^	66.49 ± 5.70^abc^
GIII	0.88 ± 0.260^b^	19.35 ± 1.73^ab^	7.22 ± 0.26^bc^	28.43 ± 1.75^bc^	106.2 ± 19.02^b^
GIV	0.74 ± 0.17^b^	22.91 ± 1.39^b^	7.57 ± 0.55^c^	32.59 ± 1.38	101.52 ± 11.22^b^
GV	0.73 ± 0.11^b^	22.39 ± 0.67^b^	7.58 ± 0.27^c^	32.51 ± 1.03^c^	112.72 ± 15.90^bc^

^a-c^Values within columns with different superscripts differ significantly by ANOVA (*P* ≤ 0.05). GI = Lantadenes @ 24 mg/kg bw; GII = Lantadenes @ 18 mg/kg bw; GIII = Lantadenes @ 12 mg/kg bw; GIV = Lantadenes @ 6 mg/kg bw; GV = control group, *n = 6*

**Table 5:** Lipid peroxidation, catalase, superoxide dismutase, reduced glutathione, and protein values (Mean ± SE) in tissue homogenate of kidneys during sub-chronic *L. camara* toxicity in guinea pigs

Group	LPO (μM of MDA/g wet tissue)	Catalase (K/mg protein)	Superoxide dismutase (U /mg protein)	Reduced glutathione (nM/ g of wet tissue)	Protein (mg/dl)
GI	1.78 ± 0.34^a^	12.83 ± 0.46^a^	3.94 ± 0.16^a^	20.12 ± 1.92^a^	68.46 ± 4.54^a^
GII	1.24 ± 0.20^ab^	15.22 ± 1.14^ab^	4.46 ± 0.31^a^	25.54 ± 1.78^ab^	74.64 ± 7.42^a^
GIII	0.92 ± 0.21^b^	15.78 ± 1.48^ab^	5.23 ± 0.50^ab^	25.71 ± 1.66^ab^	95.66 ± 16.52^ab^
GIV	0.68 ± 0.13^b^	18.52 ± 2.64^ab^	5.19 ± 0.33^ab^	30.57 ± 2.04^b^	116.92 ± 11.3^b^
GV	0.64 ± 0.09^b^	20.31 ± 2.21^b^	6.76 ± 0.66^b^	31.45 ± 0.98^b^	118.19 ± 12.65^b^

^a-b^Values within columns with different superscripts differ significantly by ANOVA (*P* ≤ 0.05). GI = Lantadenes @ 24 mg/kg bw; GII = Lantadenes @ 18 mg/kg bw; GIII = Lantadenes @ 12 mg/kg bw; GIV = Lantadenes @ 6 mg/kg bw; GV = control group, *n* = 6

### Gross and histopathological evaluation

**Fig. 3:**
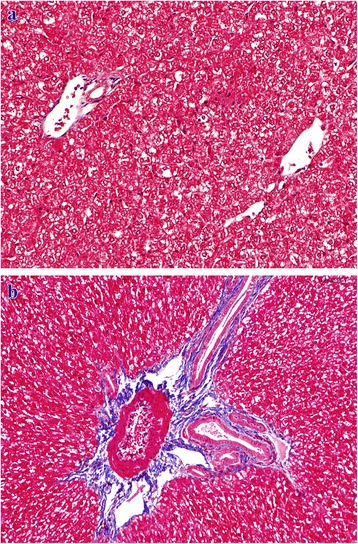
**a** Liver (control, GV): Normal liver. MSTx100. **b** Liver (GI, lantadenes, 24 mg/kg bw): Bile duct proliferation and lymphatic distention along with mild peribiliary and periportal fibrosis. MSTx200

**Fig. 4:**
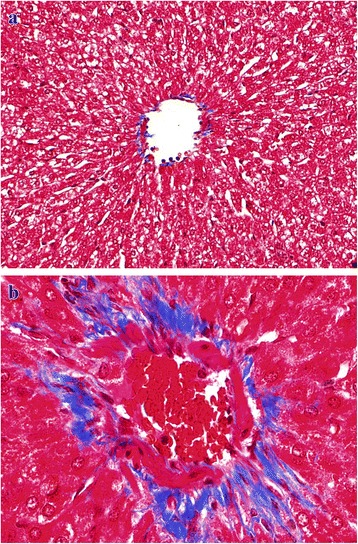
**a** Liver (control, GV): Normal liver without centrilobular fibrosis.MSTx100. **b** Liver (GI, lantadenes, 24 mg/kg bw): Mild centrilobular fibrous tissue proliferation. MSTx200

**Fig. 5:**
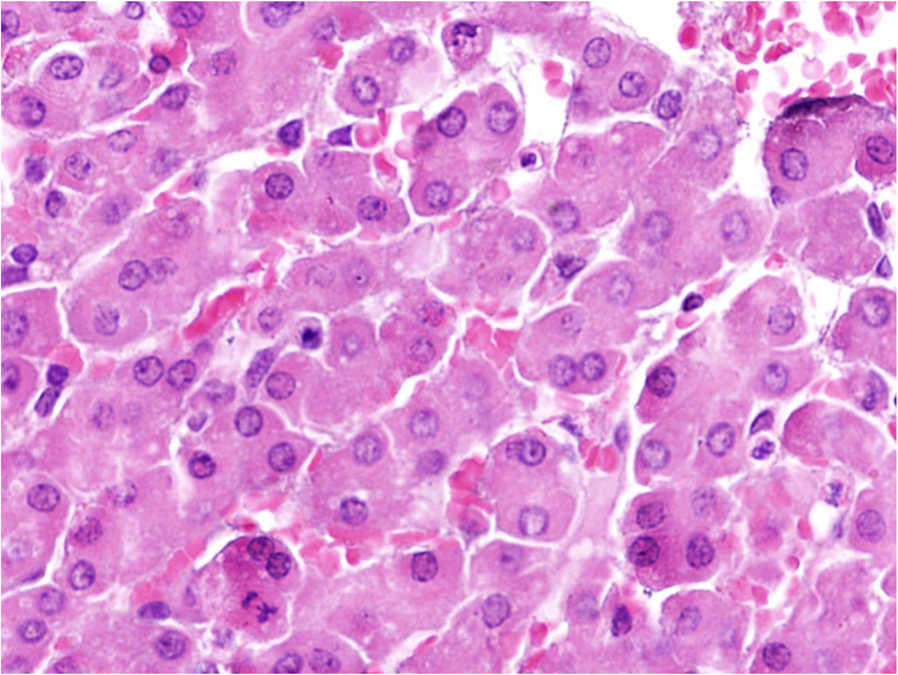
Liver (GI, lantadenes, 24 mg/kg bw): Binucleated hepatocytes along with mitotic figures indicating regenerative changes. HEx200

**Fig. 6:**
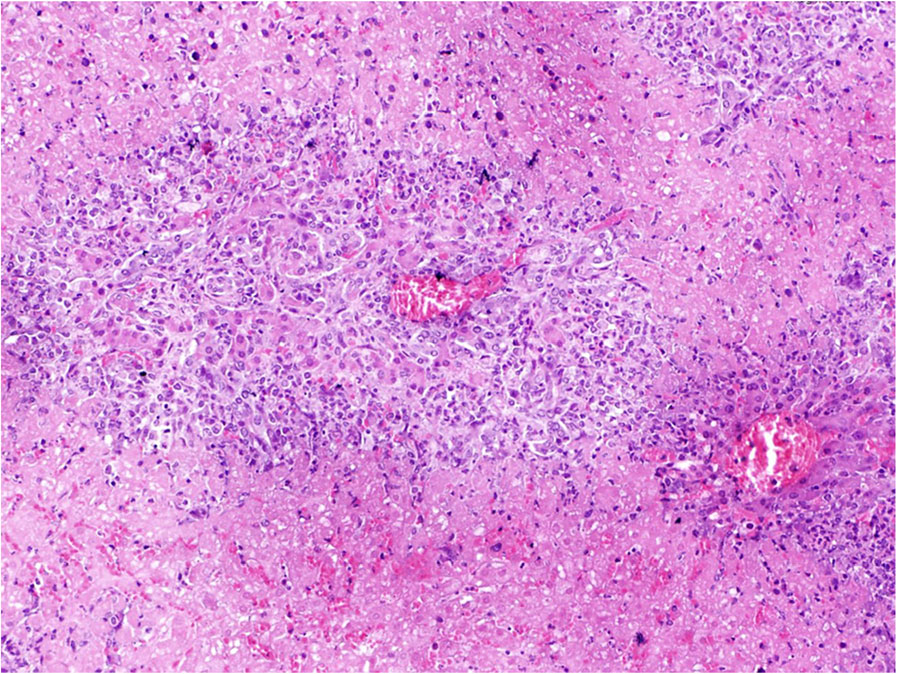
Liver (GI, lantadenes, 24 mg/kg bw): Severe multifocal necrotic areas along with inflammatory cells and ducutular reaction in hepatic parenchyma. HEx100

Grossly, the liver of animals of groups I (24 mg/kg bw) and II (18 mg/kg bw) showed presence of variable areas of necrosis which were embedded deep and margins of liver lobes were friable. Group I histopathologically showed lymphatic distension, mild bile duct proliferation, collagen fibres deposition in peribiliary, periportal (Fig. 3a, b) and centrilobular (Fig. 4a, b) regions which was confirmed by MST staining. The results of MST staining showed the presence of mild to moderate collagen fibres deposition around central vein and portal triad. Binucleated hepatocytes, a few mitotic figures and increased Kupffer cell activity which could be assessed by their increased number and size were noticed (Fig. 5). Besides these, at certain places, ductular reaction, focal areas of karyomegaly, karyorrhexis, and degenerative changes along with infiltration of heterophils were evident in the hepatic parenchyma, which were indicative of coagulative necrosis (Fig. 6). While in certain areas, diffuse mononuclear cells (mainly lymphocytes and macrophages) infiltration was also seen. Almost similar changes were seen in the animals of group II. But the intensity of degenerative changes, necrosis and apoptosis was more pronounced in group I as compared with group II. Although scattered areas of necrosis were evident in the animals of groups I and II, no relevant level of significance was observed. However, they showed significant change in the lesion score values, namely, peribiliary and periportal fibrosis, bile duct proliferation, increased Kupffer cell activity, inflammatory changes, apoptotic changes, degenerative changes and regenerative changes shown by binucleated hepatocytes and mitotic figures as shown in Table 6. The animals of other groups also showed these changes but were of lesser intensity as compared to groups I and II. The lesion score for histopathological changes in liver has been tabulated in Table 6.

**Table 6:** Lesion score (Mean ± SE) for histopathological changes in liver during sub-chronic *L. camara* toxicity in guinea pigs

Group	Necrosis	Fibrosis	Bile duct proliferation	Increased Kupffer cell activity	Inflammatory change	Apoptosis	Degenerative change	Regenerative change
GI	2.00 ± 0.26^a^	1.67 ± 0.21^b^	1.50 ± 0.34^b^	2.50 ± 0.22^c^	1.83 ± 0.31^b^	1.33 ± 0.21^bc^	1.67 ± 0.21^b^	2.17 ± 0.31^b^
GII	1.33 ± 0.33^ab^	1.50 ± 0.22^b^	1.33 ± 0.21^b^	1.67 ± 0.21^bc^	1.67 ± 0.21 ^b^	1.83 ± 0.17 ^c^	1.33 ± 0.21^b^	1.67 ± 0.21 ^b^
GIII	1.17 ± 0.31^ab^	0.83 ± 0.17^ab^	1.00 ± 0.26^ab^	1.17 ± 0.17^abc^	1.17 ± 0.31^ab^	1.00 ± 0.26^abc^	1.00 ± 0.26^ab^	1.33 ± 0.21^ab^
G1V	0.67 ± 0.21^ab^	0.67 ± 0.21^ab^	0.50 ± 0.22^ab^	0.67 ± 0.21^ab^	0.67 ± 0.21^ab^	0.33 ± 0.21^ab^	0.83 ± 0.31^ab^	1.00 ± 0.26^ab^
GV	0.00 ± 0.00^a^	0.00 ± 0.00^a^	0.00 ± 0.00^a^	0.00 ± 0.00^a^	0.00 ± 0.00^a^	0.00 ± 0.00^a^	0.00 ± 0.00^a^	0.00 ± 0.00^a^

^a-c^Values within columns with different superscripts differ significantly by ANOVA (*P* ≤ 0.05). GI = Lantadenes @ 24 mg/kg bw; GII = Lantadenes @ 18 mg/kgbw; GIII = Lantadenes @ 12 mg/kg bw; GIV = Lantadenes @ 6 mg/kg bw; GV = control group, *n = 6*

**Fig. 7:**
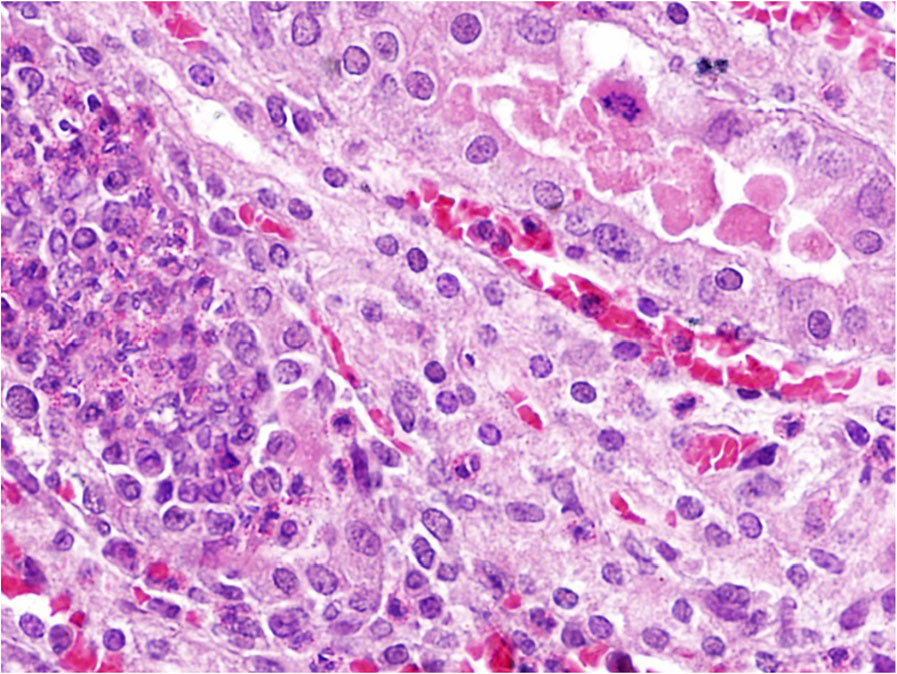
Kidney (GI, lantadenes, 24 mg/kg bw): Formation of hyaline and granular casts in tubular lumens. HEx200

**Fig. 8:**
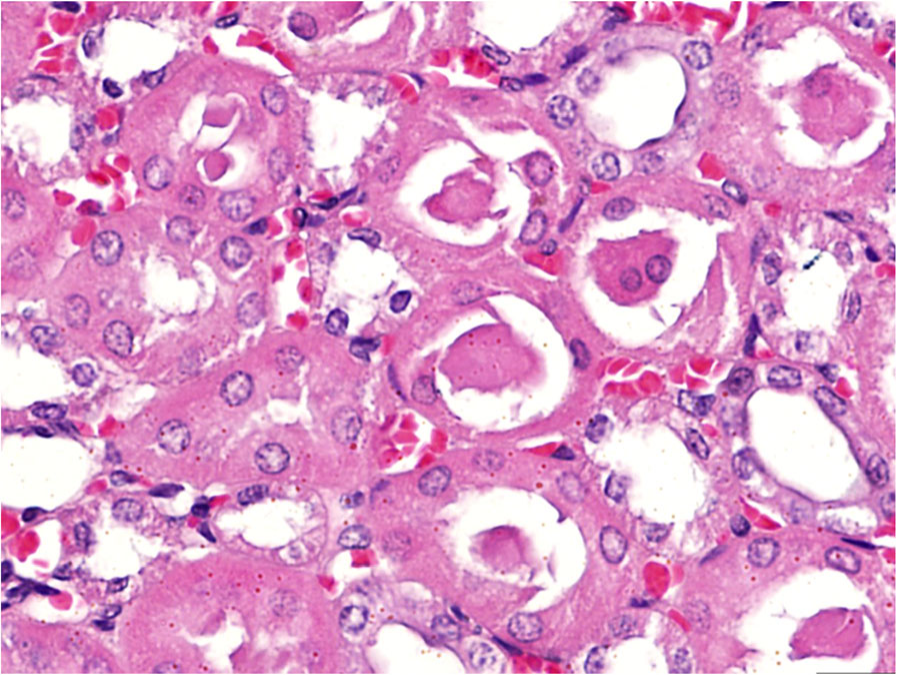
Kidney (GII, lantadenes, 18 mg/kg bw): Tubules infiltrated with epithelial and hyaline casts. HEx400

**Fig. 9:**
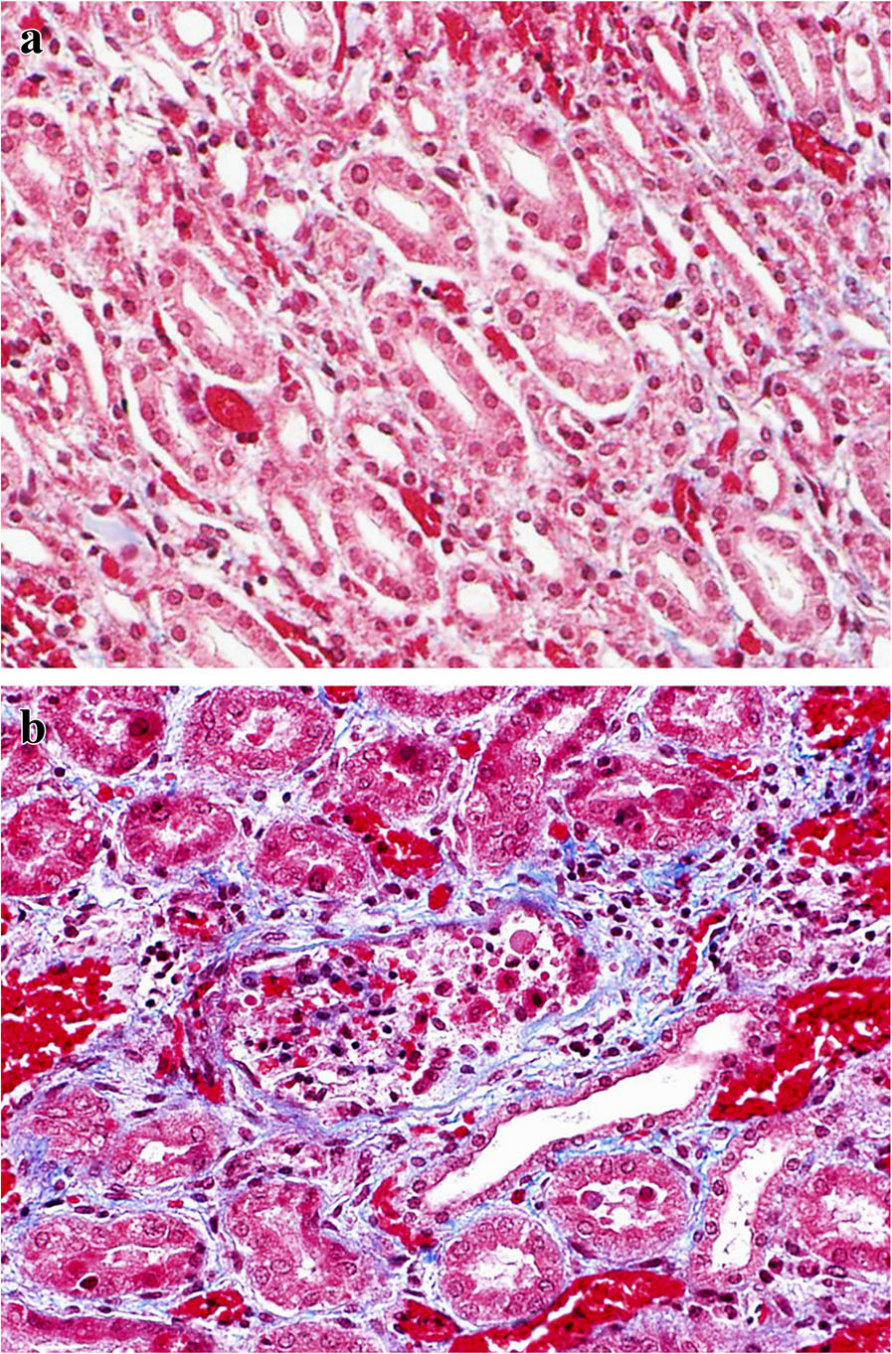
**a** Kidney (control, GV): Normal, without any peritubular and periglomerular collagenous fibrous tissue proliferation. MSTx100. **b** Kidney (GI, lantadenes, 24 mg/kg bw): Mild to moderate peritubular and periglomerular collagenous fibrous tissue proliferation. MSTx200

**Fig. 10:**
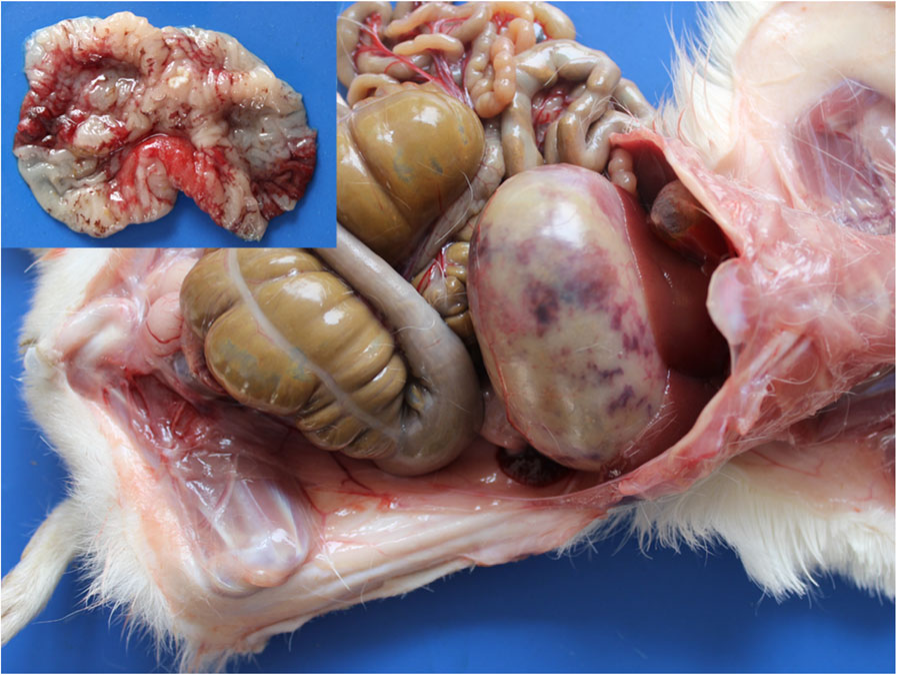
Stomach (GI, lantadenes, 24 mg/kg bw): Areas of haemorrhages on the serosa and mucosal surface (inset)

**Fig. 11:**
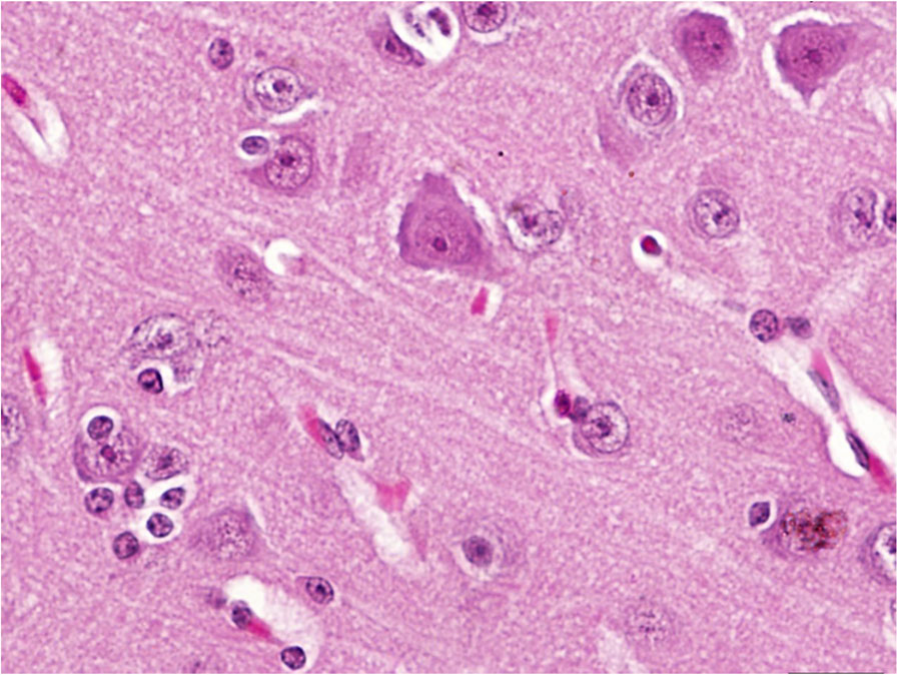
Brain (GII, lantadenes, 18 mg/kg bw): Cerebral cortex showing neuronal satellitosis. HEx400

In kidneys, the gross lesions were minimal and on histopathological examination, the animals of groups I and II showed severe degenerative and focal necrotic changes in tubules along with the infiltration of leucocytic cells (Fig. 7). In addition, there was marked deposition of hyaline and epithelial casts (Fig. 8) in the renal tubular lumens of animals of groups I and II. The periglomerular and peritubular fibrosis was also observed in sub-chronic lantadene toxicity mainly in the animals of groups I and II which was further confirmed by MST staining (Fig. 9a, b). The lesion score for histopathological changes in kidneys has been tabulated in Table 7. The gall bladder of animals of groups I and II was grossly distended. On histopathology, the gall bladder showed mild fibrosis along with inflammatory cells infiltration. The mesenteric lymph nodes (MLNs) of most of the treatment groups showed congestion of medullary region as compared to control group. Histopathologically, the MLNs of animals of group I showed lymphocytic depletion while other groups did not exhibit such changes. Grossly, the stomach of animals of groups I and II showed diffuse haemorrhages (Fig. 10) and similar lesions along with infiltration of MNCs (mainly lymphocytes and macrophages) on histopathological examination were seen. Brain of most of the treated group animals did not show any gross changes. However, on histopathology, cerebral cortex revealed mild increase in the perivascular space, neuronal shrinkage and focal areas of mild glial cell proliferation around degenerated neurons i.e. neuronal satellitosis (Fig. 11) was evident as compared to control group. This was suggestive of toxic effect even on the nervous system during sub-chronic exposure to lantadenes. The adrenal glands also showed scattered pyknotic nuclei in the cortical region during lantadene toxicity.

**Table 7:** Lesion score (Mean ± SE) for histopathological changes in kidneys during sub-chronic *L. camara* toxicity in guinea pigs

Group	Necrosis	Fibrosis	Degenerative change	Inflammatory change	Hyaline casts formation	Epithelial casts formation	Apoptosis
GI	2.33 ± 0.21^c^	2.00 ± 0.26^c^	2.50 ± 0.22^c^	2.17 ± 0.31^b^	2.83 ± 0.17^c^	2.67 ± 0.21^c^	1.67 ± 0.21^b^
GII	2.00 ± 0.26^bc^	1.50 ± 0.22^bc^	2.50 ± 0.22^c^	1.83 ± 0.31^b^	2.50 ± 0.22^bc^	2.33 ± 0.21^bc^	1.00 ± 0.37^ab^
GIII	1.17 ± 0.31^abc^	0.83 ± 0.17^abc^	1.33 ± 0.21^abc^	1.17 ± 0.31^ab^	1.50 ± 0.34^abc^	1.17 ± 0.31^abc^	0.83 ± 0.17^ab^
G1V	0.67 ± 0.21^ab^	0.33 ± 0.21^ab^	0.67 ± 0.21^ab^	0.83 ± 0.31^ab^	0.50 ± 0.22^ab^	0.50 ± 0.22^ab^	1.00 ± 0.26^ab^
GV	0.00 ± 0.00^a^	0.00 ± 0.00^a^	0.00 ± 0.00^a^	0.00 ± 0.00^a^	0.00 ± 0.00^a^	0.00 ± 0.00^a^	0.00 ± 0.00^a^

^a-c^Values within columns with different superscripts differ significantly by ANOVA (*P* ≤ 0.05). GI = Lantadenes @ 24 mg/kg bw; GII = Lantadenes @ 18 mg/kg bw; GIII = Lantadenes @ 12 mg/kg bw; GIV = Lantadenes @ 6 mg/kg bw; GV = control group, *n* = 6

## Discussion

*Lantana camara* introduced as an ornamental shrub has become a threat to our livestock and biodiversity [2]. This plant is capable to cause mortality in ruminant as well as non-ruminant species. Among the non-ruminants, guinea pigs are the most susceptible [17] species and therefore have been used as a model in the present study. This weed leads to hepatotoxicity and photosensitization in grazing animals and has allelopathic effect on other vegetation [2]. Major toxic components present in this plant are lantadenes [18, 19]. In the present study, LA and LB content in the leaves was estimated to be 49.18% and 13.09%, respectively by reverse phase-HPLC.

The orally administered lantadenes can be absorbed from stomach, small as well as large intestine. But small intestine is the most important route. The absorption is affected by presence of ingesta in the intestine [20]. Therefore, the animals in the present study were provided with feed and water one hour after the oral administration of lantadenes. After absorption, the transportation of toxin to the liver occurs via portal route and bile has no effect in this absorptive mechanism [2]. For the maintenance of effect of toxin, continuous absorption of toxin is required. This toxin can lead to ruminal stasis in cattle due to inhibitory neural impulses arising from damaged liver after 4–6 h of administration [21, 22]. In the present study, there was a significant decrease in weekly body weights of the animals of group I, while decrease in the body weights of animals of groups II, III and IV was not significant as compared to control. After absorption, lantadenes mainly acts on bile canalicular plasma membrane (CPM) which is the main target of its action in the hepatocytes [23, 24]. The biliary compounds from the blood are mainly taken to sinusoidal membrane and from there stored and metabolized by hepatocytes and ultimately secreted by CPM. The damaged CPM is not capable to secrete the bile thus leads to impaired hepatobiliary excretion and thereby intrahepatic cholestasis [23, 24]. The cholestasis is accompanied by dilated bile duct canaliculi, microvilli loss, alteration in enzymes and jaundice [25, 26]. Jaundice has been reported in acute and sub-acute lantadene toxicity and its development depends upon the dose of lantadenes administered and action of lantadenes on biliary secretions, which leads to cholestasis and thereby jaundice [2, 27, 28]. However, ictericity was not observed in sub-chronic toxicity as the biliary mechanism is least altered and can be compared with minimal effect of lantadenes on the values of ALP, which gives a clear evidence for no development of cholestatic mechanism and ictericity in animals. But the presence of lower doses of this toxin in circulation might be capable to cause more sub-chronic changes and degenerations, which were minimal in previous studies on acute and sub-acute lantadenes exposure [2, 7].

The total platelet counts of groups I and II were significantly decreased as compared to control. The possible mechanism for this can be the haemorrhages caused by the action of lantadenes in stomach leading to anaemia. The significant decline in the values of Hb in the animals of group I might be because of defective haemopoietic mechanism, whereas decrease in the values of PCV indicated shrinkage in the size of erythrocytes as a result of lantadenes intoxication as studied earlier [29]. In one of the studies, transient decrease in PCV, TEC, Hb values and increase in neutrophil count has been reported in cattle and buffaloes [30, 31]. Increased levels of ALT, AST and ACP of animals of groups I, II, III and IV indicated hepatic damage as these enzymes leaked out from the hepatocytes into blood due to tissue damage. However, this increase was significant only in group I, which was the highest dose group (24 mg/kg bw) and the elevation in this group could be correlated with marked histopathological changes. A slight increase in the values of ALP was observed in the animals of groups I and II, but this elevation was not statistically significant. The increase in the level of ALP is often associated with profound hepato-biliary injury and cholestasis. In present study, non-significant elevation in ALP levels indicates less significant involvement of biliary system in sub-chronic lantadenes toxicity. The changes in the liver specific serum enzymes indicated significant hepatic damage and were well supported by histopathological alterations in the liver. The previous study on sub-acute lantadene toxicity in guinea-pigs showed an increase in the values of ALT, AST, and ALP while no significant increase was seen in ACP levels [7]. Sharma and Sharma [32] reported that the oral administration of LA to guinea pigs elicited an increase in activities of ALT, AST, ALP and ACP. There was no significant change in the total bilirubin levels in sub-chronic lantadene toxicity. Hence, the sub-chronic oral lantadene administration did not lead to the development of icteric subcutaneous tissues and mucus membranes in the experimental animals. However, in a previous study, the total bilirubin levels were significantly elevated at high doses (100 and 50 mg/kg bw) leading to icteric subcutaneous tissue and renal medulla. The creatinine levels were also found to increase in sub-acute lantadene toxicity (25 mg/kg bw). The lower doses of lantadenes (25 and 12.5 mg/kg bw) were supposedly the main cause for renal damage due to cumulative toxicity [7]. In the present study, the changes in the kidneys were more pronounced than in liver while in sub-acute lantadene toxicity study, hepatotoxic effects were more pronounced [7]. The possible reason for this could be the regeneration of hepatocytes at lower doses of lantadenes used in the present study. The concept of regeneration was supported by presence of mitotic figures and binucleated hepatocytes. A decrease in the values of protein, increase in values of cholesterol and cholesterol: phospholipid ratio was seen on oral administration of lantadenes [33]. In present study also, the values of proteins were significantly decreased in animals of groups I, II and III as compared to group IV and control.

Malondialdehyde (MDA), the main oxidative degradation product of lipid-peroxidation, functions as a marker of oxidative injury of cellular membranes [34]. An increased MDA level in liver suggests enhanced lipid peroxidation leading to tissue damage and failure of antioxidant mechanism to prevent formation of excessive free radicals. LPO was exhibited by almost all the tissues in lantana poisoning in the order adrenals> liver> kidneys> heart> lungs> testes> brain [35]. The present study showed significant increase in the values of LPO in liver and kidney homogenates mainly in animals of group I. The SOD levels were declined in liver and kidneys of animals of group I and II at a significant level as compared to groups III and IV. Reduced glutathione (GSH) is one of the most abundant non-biological antioxidant present in liver [36]. GSH is a key component of overall antioxidant defense system that protects cell from deleterious effects of reactive oxygen species. In the present study, GSH levels of animals of groups I and II were significantly decreased as compared to control group. However, in sub-acute lantadene toxicity, there was no significant decrease in the values of GSH [7]. Reduced glutathione values were also found to decrease in kidneys of animals of group I as compared to control. There was a significant reduction in enzymatic antioxidants including SOD and catalase which are essential for endogenous antioxidative defense system to scavenge reactive oxygen species and maintain cellular redox defense [37]. In the present study, the catalase levels were significantly lower in liver as well as kidneys of animals of group I and this decrease in catalase levels was also observed in sub-acute lantadene toxicity [7]. The protein levels of liver homogenate of animals of group I was significantly decreased as compared to other groups. There was a significant reduction in the values of protein in kidney homogenate of animals of groups I and II while this decrease was not significant in other groups. In earlier study on lantadene toxicity, there was an increase in protein values in liver homogenate, while no significant difference was seen in kidneys as compared to control [7]. In the present study, it has been observed that the alteration in antioxidative enzymatic system was mainly recorded in groups I (24 mg/kg bw) and II (18 mg/kg bw) as compared to groups III, IV and V. These alterations were well supported by histopathology, special staining, oxidation stress determination and biochemical changes in liver as well as in kidneys.

Lantadenes mainly damages the peripheral parenchymal cells of liver while cells around central vein are normal [2]. Liver shows variable lesions in acute and sub-acute lantadene toxicity ranging from swelling, pale yellow and fragile appearance, with diffuse areas of necrosis and haemorrhagic streaks. The kidneys were yellowish in colour [2, 7, 38]. However, in the present study, the various doses of lantadenes did not produce severe characteristic gross lesions in liver and kidneys. The liver of animals of groups I and II exhibited a few necrotic foci on parenchyma and pale kidneys. The liver and kidneys of animals of groups III and IV did not show any significant gross changes as compared to control. However, the lesions in liver and kidneys were well evident on histopathological examination.

On histopathology, the liver of animals of groups I and II showed focal areas of hepatocytic necrosis. The nuclear changes included pyknotic nuclei, karyomegaly, karyorrhexis and dissolution of nuclei in the hepatocytes which also supports that the oxidative damage could be one of the mechanisms for hepatocellular injury [39]. In the present study, infiltration of MNCs admixed with heterophils, peribiliary and periportal fibrosis, bile duct proliferation, binucleated hepatocytes, mitotic figures, apoptotic changes and increased Kupffer cell activity was observed. Binucleated hepatocytes and mitotic figures (1–3 cells/high power fields) are important consequences of hepatocytic injury and chromosomal hyperplasia. These changes are often appreciated in the cells which are undergoing regeneration [40]. Kupffer cells are often associated with liver injury and hepatocellular necrosis and often implicated as a cause of TGF-beta production required for the transformation of stellate cells into myofibroblasts and ultimately leads to fibrosis. The increased number and activity of Kupffer cells indicated the defence mechanism of detoxification associated with hepatic injury as studied earlier [41]. The remaining treatment groups also showed similar lesions but were of lesser severity or absent in some animals. The fibrotic changes around periportal and peribiliary regions were evident in the liver of animals of groups I and II which were further confirmed by MST as well as seen in previous study on sub-acute lantadene toxicity [7]. In sub-acute lantadene toxicity study, the liver showed large areas of coagulative necrosis, heterophilic infiltration, biliary fibrosis, haemorrhages and engorgement. The collagen tissue deposition in liver of these animals was confirmed by MST [7]. In the present study, the renal lesions were more characteristic which included marked degenerative changes, necrosis, hyaline and epithelial casts, apoptosis, periglomerular and peritubular fibrosis. The degree of fibrosis was further confirmed by MST. Similar fibrotic lesions in kidneys were also seen in sub-acute toxicity of lantadenes in guinea pigs where collagen tissue deposition was also confirmed by the same special stain [7]. The severity of collagen tissue deposition in the kidneys was more pronounced in sub-chronic toxicity. Our study on sub-chronic exposure to lantadenes is also supported by a similar kind of sub-chronic toxicity trial of 90 days, where animals were administrated with methanolic extract of *Rhaphidophora decursiva* [42]. On similar lines as of our study, no changes were evident grossly, while on histopathology the changes included increased Kupffer cell activity, karyomegaly and karyorrhexis in the liver and there was formation of cellular casts and pyknotic cells in kidneys [39, 43].

In the present study, the gall bladder was grossly distended. However, collagen tissue deposition was noticed on histopathological examination which was further confirmed by MST staining. In earlier acute and sub-acute lantadene toxicity studies, the contents of gall bladder were found to be inspissated, yellowish, opaque, thick and tarry and fibrous tissue formation was not evident on histopathology [2, 7]. Grossly the MLNs of all the treatment groups showed congestion in medulla as compared to control. Lantadenes are capable to cause immunosuppression by reducing both the cellular and humoral immune system as observed in sheep [44]. In the present study, MLNs of animals of group I showed lymphocytic depletion which indicated immunosuppressive nature of lantadenes. The brain of treatment groups on histopathology showed mild glial cells proliferation around a few degenerated neurons as compared to control group, which was suggestive of toxic effect even on the nervous system during sub-chronic exposure to lantadenes. The adrenal glands showed pyknotic nuclei indicative of physiological stress induced by lantadene toxicity at lower doses.

Lantadenes produced maximum damage in kidneys followed by liver and stomach during sub-chronic exposure. The more severe changes in kidneys revealed more nephrotoxic action of lantadenes as compared to hepatotoxic action in sub-chronic toxicity study (90 days). This could be because liver has power of regeneration so it might have developed some compensatory mechanism in liver parenchymal cells or in liver canaliculi to excrete bile not leading to the development of jaundice as studied in earlier experiments on lantadene toxicity at the dose of 50 mg/kg bw. However, at lower doses of lantadenes (25 mg/kg bw) in sub-acute exposure, the associated pathological alterations like cellular degenerations and hepatic necrosis were evident as also observed in the present study on sub-chronic administration of lantadenes [7]. While the kidneys showed more pronounced damage because of persistent effect of toxins and exhibited typical signs of sub-chronic toxicity.

## Conclusions

In conclusion, the present study brought to light the finding that sub-chronic exposure to lantadenes produce a dose-dependent toxicity of lantadenes in decreasing order in the guinea pigs, with the highest dose (24 mg/kg bw) producing maximum and the lowest dose (6 mg/kg bw) producing minimum alterations in kidneys as well as in liver. The results of significant decline in the body weights, hematology, serum marker enzymes, oxidation stress levels, gross and histopathological examination and confirmation of collagen tissue by MST staining showed that the lantadenes, which are generally considered hepatotoxic, rather resulted in pronounced nephrotoxicity during sub-chronic exposure.

## Abbreviations

GSH: Reduced glutathione
LA: Lantadene A
LB: Lantadene B
LPO: Lipid peroxidation
MLN: Mesenteric lymph node
MNC: Mononuclear cells
MST: Masson’s trichome stain
SOD: Superoxide dismutase
